# Health monitoring of refugees in reception centres for asylum seekers: Decentralized surveillance network for the analysis of routine medical data

**DOI:** 10.25646/7865

**Published:** 2021-03-31

**Authors:** Rosa Jahn, Sven Rohleder, Markus Qreini, Stella Erdmann, Sukhvir Kaur, Frank Aluttis, Kayvan Bozorgmehr

**Affiliations:** 1 Section for Health Equity Studies and Migration, Department of General Practice and Health Services Research, University Hospital Heidelberg, Heidelberg, Germany; 2 Department of Population Medicine and Health Services Research, School of Public Health, Bielefeld University, Bielefeld, Germany; 3 Institute of Medical Biometry and Informatics (IMBI), University Hospital Heidelberg, Heidelberg, Germany

**Keywords:** HEALTH MONITORING, SURVEILLANCE, MIGRATION, FORCED DISPLACEMENT, ASYLUM, DISTRIBUTED COMPUTING

## Abstract

Refugees and asylum seekers living in reception centres tend to be not adequately included in population-based studies, routine medical data and official statistics. As part of the research project ‘Health and primary-care sentinel surveillance in reception- and accommodation-centres for asylum-seekers in Germany’ (PriCare), a health-monitoring approach was developed for the secondary use of routine medical data from on-site outpatient clinics in reception centres. To this end, a software application (Refugee Care Manager, RefCare©) for the digitisation and harmonisation of medical records was designed and implemented in reception centres in three German federal states. The approach of distributed computing in a surveillance network allows for the decentralised, harmonised analysis of the routine medical data stored in RefCare© in a manner that fully complies with data protection regulations and circumvents the need for centralised data storage. RefCare© provides an integrated surveillance feature that enables analyses of 64 indicators on population, morbidity, healthcare processes and quality of care to be undertaken across multiple facilities. This article describes the conceptual and practical approach and the technical procedures put in place to do so, and provides examples of the results that have been gained so far.

## 1. Introduction

The federal health monitoring is tasked with compiling robust data on health, health risks, and healthcare provision for the population in Germany from a broad range of sources, and making this information available to decision-makers and the general public. However, asylum seekers and refugees are insufficiently included in health-related data collection in Germany and other European countries [1]. This shortcoming also applies to routine medical data, defined as all personal data stemming from health and social services that are primarily collected for routine administrative purposes (cf. [4]). These data provide an essential basis with which to study morbidity and the utilisation of medical services [2–4].

In Germany, asylum seekers are not provided with statutory health insurance and commonly receive medical services on-site in the reception centres, with infrequent referrals to specialist outpatient medical practices. They thus receive healthcare largely outside of regular care provision structures and associated routine medical data sources. The centralisation of the asylum process over the last five years has increased the length of time that asylum applicants live in reception centres. Particularly individuals from ‘safe countries of origin’ may now remain in reception centres for the duration of their entire asylum process. Moreover, during the first 18 months of their stay in Germany, asylum seekers are only entitled to a limited range of medical care. The electronic health card, which facilitates access to healthcare in Germany and enables data on diagnoses and healthcare provision to be recorded digitally, is also not consistently made available [5]. Spatial, legal and administrative differences, therefore, mean that healthcare is provided to asylum applicants within structures that are inadequately linked to information sources such as routine data (e.g. data from health insurers) [6]. Some federal states provide asylum applicants with an electronic health card, and the healthcare they receive is therefore included in the routine data collected by health insurance providers [7]. However, as these data are only available on a quarterly basis and are primarily gathered for accounting purposes, they are unsuitable for both target-group specific monitoring, and the timely and ongoing surveillance of the health of and healthcare provided to asylum applicants in reception centres, in particular.

**Info box**

The forms of accommodation used to house asylum seekers at the state level can be divided into registration and reception centres. When asylum applicants are first registered, they tend to be placed in registration centres, which only provide for a short-term stay. In contrast, reception centres provide medium-term accommodation, and asylum applicants may remain in these facilities for up to 18 months. In fact, depending on their country of origin, some applicants remain in reception centres accommodation during the entire application process and until a decision has been made on whether to transfer them to a district-level facility.

These facilities can be further distinguished from the accommodation centres provided at the district level, which tend to vary in size and are a temporary measure. Depending on the outcome of an asylum application, applicants would then be transferred to private housing (see also the article Monitoring the health and healthcare provision for refugees in collective accommodation centres in this issue of the Journal of Health Monitoring).

For the sake of simplicity, these institutions are all referred to here as ‘reception centres’ or ‘facilities’ unless a particular type of facility is being referred to.

With this is mind the development of setting-specific systems for the standardised recording, regular analysis and communication of data about the health of and healthcare provided to asylum applicants in reception centres is an important means of raising awareness about the health needs of this population group (Info box).

Medical records from outpatient clinics in reception centres constitute an important data source on the health of refugees, but timely monitoring across all facilities requires standardised and, preferably, electronic medical records. Due to the lack of national and regional guidelines on healthcare provision and medical records in reception centres, however, the situation is characterised by a high degree of fragmentation [8]. The on-site medical services use different electronic medical records, which are often unsuitable for the specific setting of reception centres, and some even rely on paper-based index card systems [9]. The lack of compatible or digital medical records not only has a negative impact on the quality of care caused by communication difficulties for health providers inside and outside of the facilities [10, 11]. It also means that routine medical data from clinics in reception centres are not readily available for analysis and health reporting [10, 12].

The project ‘Health and primary-care sentinel surveillance in reception- and accommodation-centres for asylum-seekers in Germany’ (PriCare, duration: 2016–2020), which was funded by the German Federal Ministry of Health, was established to improve the quality of medical records in reception centres and to set up a system that can be used to routinely monitor the health of and the healthcare provided to asylum seekers and refugees [9]. The project developed and implemented a tailored software (RefCare©) to digitise and harmonise medical records in reception centres. In addition, in cooperation with medical service providers, the project developed a means of performing regular, automated and realtime statistical analyses of the local routine data using pre-defined indicators. This article describes the conceptual and practical approach and the technical procedures that were put in place to do so, and provides examples of the results that have been gained so far.

## 2. Methodology

### 2.1 Software development and implementation

Reflecting the legal requirements for medical records as well as experiences gained from working with healthcare providers in reception centres for asylum seekers, we developed a prototype for the electronic medical records system RefCare©. The prototype provided the basic functions found in typical electronic medical records. Functionalities that were less relevant for care provision in reception centres, such as the accounting of medical services, were disregarded while others, such as vaccinations and multilingual communication, were added. The prototype underwent usability tests with eight doctors working in reception centres [9]. Usability issues raised by these tests were discussed with the software development team and addressed accordingly.

**Figure 1:**
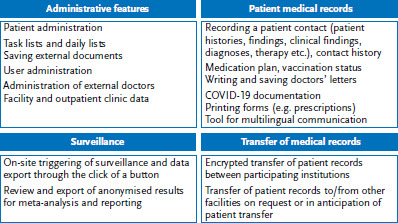
Current overview of the functions available in RefCare© (as of October 2020) Source: PriCare network, Heidelberg University Hospital

A pilot version was tested as of October 2017 in a clinic in a large reception centre in Bavaria. Feedback was systematically logged and recorded, before being checked for feasibility by the project and development team; ideas were then prioritised and built into the software wherever possible. Additional test sites followed in 2018 in Bavaria and Hamburg, with the collection of feedback from users and iterative software development continuing as a long-term process that went beyond the initial pilot phase. During this period, the software and the implementation process were further adapted and tailored for use in reception centres (for details, see [9]). This process included that the software supported patient files to be sent between reception centres and healthcare providers for further treatment in a manner that complied with data protection regulations. In addition, a multilingual patient interface and, in March 2020, a module for recording the screening and testing for SARS-CoV-2, and treatment of patients with COVID-19, were also added (Figure 1). Interfaces with existing medical software systems are in the planning stages (as of January 2021).

### 2.2 Distributed computing network

**Figure 2:**
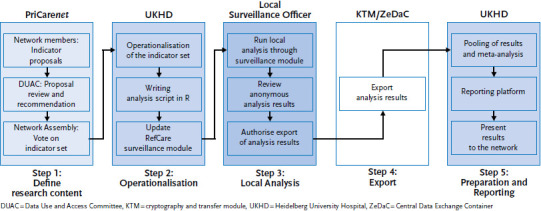
The hive approach: flow chart of distributed computing used by the PriCarenet network Source: PriCarenet network, Heidelberg University Hospital

RefCare© enables medical records to be digitised in a standardised manner, and, therefore, provides for cross-facility harmonisation of locally-stored routine data in participating facilities. In order to perform a cross-institutional analysis of this routine data, and comply with data protection regulations, a procedure was developed that enables decentralised analyses to be conducted without the need to disclose personal information to third parties (i.e. outside of the facilities themselves). This procedure is known as ‘networked distributed computing with the result of an anonymised indicator’. In the following, it is simply referred to as the ‘hive approach’ due to the large number of decentralised yet coordinated analytical processes involved (Figure 2). The hive approach to health monitoring follows five steps.

#### Step 1: Building consensus on surveillance indicators within the PriCarenet research network

The PriCarenet research network consists of representatives from facilities that use RefCare©, the authorities involved, and academic partner institutions. The network jointly develops and adapts the indicator set used in the routine health surveillance. Each member of the network can submit and provide arguments in favour of proposals for new indicators. The scientific basis, feasibility and ethics of the proposals are then assessed by the elected Data Use and Access Committee (DUAC). The committee subsequently provides a recommendation and the indicators are presented to the network’s members at the network’s general assembly, which is its decision-making body. Finally, members vote on whether to include the indicator in the set; only representatives from the clinics are entitled to vote. The aim of the DUAC is not to secure access to the data ‘from the outside’, but to select and coordinate indicators and scientific research questions for analysis at the local level.

The current health and healthcare indicator set, which has been approved by the PriCarenet network, consists of 64 indicators from the fields of population, morbidity, quality of care, healthcare process and syndromic surveillance (Table 1).

#### Step 2: Operationalisation of health and healthcare indicators

Once an indicator has been approved by the research network, it is operationalised by the project team at Heidelberg University Hospital. Operationalisation is based on the data fields available in RefCare©, particularly diagnosis (International Statistical Classification of Diseases and Related Health Problems, 10th revision, German Modification, ICD-10-GM), reason for seeking medical advice (International Classification of Primary Care, ICPC), prescriptions (Anatomical Therapeutic Chemical Classification System, ATC), vital parameters (e.g. heart and respiratory rate, fever, blood pressure), medical referral forms, and personal data (e.g. age, sex, country of origin). If necessary, software updates can be used to provide new fields, responding to challenges such as the COVID-19 pandemic. To comply with the principle of data minimization, only patient data required for the immediate care provision can be recorded.

Based on the indicator operationalisation, an analysis script is then produced in the programming language ‘R’. The script is internally validated by a second team member, using methods such as independent programming, in order to review the plausibility of its results. The script is then made available to the clinics for decentralised, automated analysis via a RefCare© software update [13].

#### Step 3: Local analysis of routine medical data using the surveillance module

In principle, the analysis of the local routine medical data is conducted by the care providers on a voluntary basis. The analysis script can be executed locally through the integrated RefCare© surveillance module by a simple click on a button (Figure 1). The script begins by anonymising the data set before calculating the results (for details about the technical process see [7]). Access to the surveillance module is only granted to authorised surveillance officers in the clinic itself. Surveillance officers are selected by the responsible staff in the facilities and they are provided with training on how to use the module. They are also given written supporting information and brief instructions. In order to ensure that the analyses are standardised, and to provide for conclusive meta-analyses, analyses are carried out for cross-facility reporting for precise monthly periods up to the last day of each month. However, facilities can also conduct additional analyses for user-defined periods for their own purposes.

Once the analyses have been completed, the results are immediately available locally. The results are saved in Excel files containing anonymised (i.e. aggregated) figures, such as absolute and relative frequencies or prevalence. Surveillance officers are also provided with an introduction and written instructions to reading and interpreting the output in Excel. This ensures that the healthcare providers have the skills needed to view the results on-site and to assess their plausibility.

#### Step 4: Encrypted export of the results

The surveillance officers then export the data for the defined surveillance periods from the facility to Heidelberg University Hospital. Exporting the results, too, is voluntary, and occurs independently of the local data analysis. The results are exported via a cryptography transfer module (Figure 2) integrated into RefCare©. This module enables the results to be sent as a data package to the Central Data Exchange Container (ZeDaC), together with details of the addressee (Heidelberg University Hospital) and the sender, before being stored in encrypted form. The PriCare project team at Heidelberg University Hospital can then automatically download the data packages stored on the ZeDaC system and transfer them to an internal surveillance server. Once data packages have been retrieved from ZeDaC they are deleted from the system.

#### Step 5: Preparation and meta-analysis of facility-specific results

In this step, the anonymised indicator results stored on the surveillance server in Heidelberg undergo automatic graphical processing using R and JavaScript, and are then displayed on a dynamic reporting platform. In this manner, both the results of the facility-specific and cross-facility meta-analyses are made available on the reporting platform. Each facility has its own login details which they can use to view their own analysis results, as well as anonymised data points from other facilities for benchmarking purposes. In addition to automated reporting via the reporting platform, further meta-analyses can be carried out across institutions and are published in regular surveillance reports without providing the names of specific facilities. In order to promote the translation of the analysis results and their utilization for care provision, the results and their possible implications are discussed at the assembly of the research network PriCarenet. This also ensures that the plausibility of the results is assessed regularly and that the indicators can be expanded and supplemented. If needed, facility-specific results can also be made available to the authorities responsible for the reception centres and to higher-level political decision-makers, either through direct access to the reporting platform, or through the healthcare provider. Cross-facility data are published by the network without reference to individual facilities and can thus inform political decision-making processes.

#### Data protection regulations

Data protection poses a major challenge to regular health monitoring across institutions, especially in the fragmented and heterogeneous structures found in care provision settings at reception centres for asylum seekers. At the same time, the workload faced by medical staff at the facilities, language issues, and the vulnerability of asylum applicants, means that it is practically impossible to obtain informed consent for research using routine medical data from each patient. However, these challenges can be overcome with the hive approach as it enables researchers to protect the sensitive, personal data of a highly vulnerable population while still conducting cross-institutional health monitoring at regular intervals. Furthermore, this approach also enables medical service providers to evaluate their routine medical data automatically and anonymously and to do so in their own facilities without the need to disclose personal data to third parties. Finally, the approach yields indicators that do not enable any conclusions to be made about specific individuals, and, therefore, the indicator results can be passed on to third parties while still complying with data protection regulations.

The hive approach has a fundamental advantage over traditional surveillance that relies on centralised databases and analyses in remote research facilities: it requires no central storage of personal data. As the results from the various clinics are available in the same format (because they are produced by a standardised script) they can nonetheless be summarised through meta-analysis and comparisons can be made between facilities. The PriCarenet network provides an essential foundation for adherence to data protection regulations, and, therefore, for the use of distributed computing/the hive approach. It ensures that facilities have a say in the analysis of their data and the contents of the routine surveillance. Moreover, the local analysis of routine data through the surveillance module as well as the decision to export data are both voluntary. As the script used to analyse a facility’s data is run on-site, analyses can therefore be justified by a health provider’s legitimate interest in undertaking in-house research for healthcare planning and quality assurance. Depending on the type of healthcare setting and the way in which data protection responsibilities are organised, the legal basis for this type of data analysis is provided by data protection laws at the state or federal level (e.g. §27 Paragraph 1 of the Federal Data Protection Act). Since the analyses are conducted for in-house research with the aim of improving healthcare provision, and because the approach respects data minimisation and guarantees patient anonymity, there is no need to seek prior consent from the patients.

### 2.3 Examples of statistical analyses

The following provides examples of facility-specific and cross-facility analyses that can be carried out automatically at regular intervals as part of the PriCare project. These examples illustrate the potential of the approach for monitoring the health of asylum seekers and refugees. The results are an excerpt of the information that can be routinely accessed via the reporting platform. The following describes both a facility-specific analysis from a sample facility for the period between 1 May 2018 and 31 August 2020 as well as a cross-facility analysis of a morbidity indicator using data from eleven facilities from the period beginning with the implementation of RefCare© until 31 October 2018. The facilities are grouped by level and include registration centres (level 1) and reception centres (level 2) at the state level as well as accommodation centres at the district level (level 3) (see also the article Monitoring the health and healthcare provision for refugees in collective accommodation centres in this issue of the Journal of Health Monitoring). Since the length of time spent in these facilities, the spectrum of morbidity, and the countries of origin differ in each facility, they are grouped by accommodation type for ease of comparability. Annex Table 1 depicts the operationalisation of the morbidity indicators included in the analysis.

#### Facility-specific analyses

These analyses include information on patient numbers and the number of times that patients have attended the clinic. They also provide an overview of the most common countries of origin in absolute patient numbers per month, in addition to stating a monthly prevalence for the morbidity indicator ‘mental and behavioural disorders’ (ICD-10 diagnoses: F00–F99) stratified by age and sex. Furthermore, morbidity profiles are generated for individual facilities using 29 morbidity indicators. This enables a period prevalence to be calculated for each indicator and each institution. When calculating prevalences, the total number of cases for each indicator serves as the numerator, with the total number of people acting as the denominator. In principle, the total number of people living in the facilities could also be used as the denominator. However, these statistics are not recorded directly in RefCare©, and, instead, are collected through a separate survey undertaken in each of the facilities participating in the network on the 15th of each month, stratified by age group and sex. As different facilities achieve different levels of completeness, the total number of people treated has proven a more reliable denominator.

#### Cross-facility analyses

The weighted, pooled prevalence and the 95% confidence interval for the morbidity indicator ‘mental and behavioural disorders’ are provided here, stratified by age and sex. The estimator is calculated using a meta-analysis via a random effects model, in which variance between facilities is accounted for as a random variable. The facility-specific and the pooled estimators are depicted using a forest plot, stratified by age (children up to 18 years, adults aged 18 or above) and sex (female, male).

## 3. Results

Since the beginning of the project and the implementation of RefCare© in a pilot facility, the software has been implemented in 29 institutions in Baden-Württemberg, Bavaria and Hamburg. Due to facility closures and changes in service provider, as of 2 October 2020, the software is currently used by 24 facilities in these federal states.

The PriCare project successfully developed and implemented the infrastructure required for distributed computing, the PriCarenet research network itself, the indicator set, the analysis script and the surveillance module. Each of the 24 facilities can perform automated analyses and utilise the results for on-site planning and reporting purposes. The results particularly enable service providers to meet their sometimes contractually agreed reporting obligations with the authorities and thus directly facilitate their work and improve communication. Sharing facility-specific and cross-facility results within the research network has proven fruitful and contributes to more robust interpretations of the results, enables the indicator set to be adapted, and encourages people to share good practices in the provision of medical care in reception centres.

### 3.1 Example results from one facility

**Figure 3:**
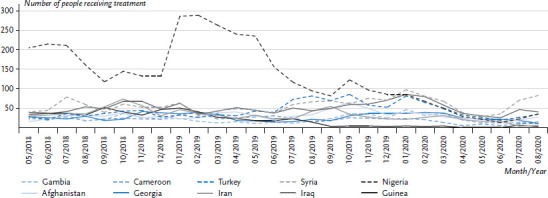
The ten most common countries of origin for people who received treatment over the entire period (absolute monthly patient numbers, n = 11,579) Source: PriCarenet network, Heidelberg University Hospital

A total of 11,579 patients were recorded in RefCare© in the facility in question between May 2018 and August 2020. The patients comprised 9,853 adults (85.1%), of whom 3,980 (40.4%) were female, 5,870 (59.6%) were male, and 3 (0.03%) were missing information on sex. 1,726 were children and adolescents (14.9%), of whom 791 (45.8%) were female, 928 (53.8%) were male, and 7 (0.4%) were missing information on sex. Overall, a total of 38,171 patient contacts were recorded. The mean number of contacts per patient was 3.5 for adults; 2.3 for children; 3.9 for female patients; and 2.9 for male patients. The ten most frequent countries of origin among all patients in the observation period per month are shown in Figure 3.

**Figure 4:**
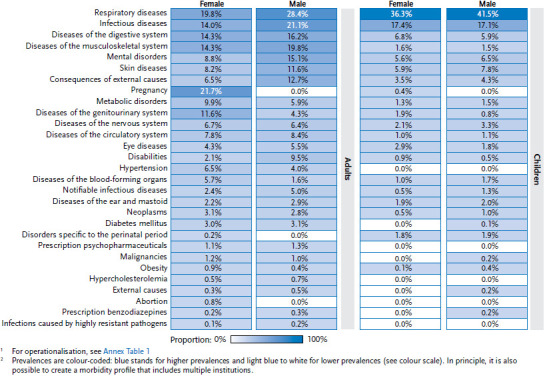
Morbidity profile of the sample facility depicting the prevalence of morbidity indicators by age and sex (as a percentage of people treated^1^), (adults: n = 3,980 female, n = 5,870 male; children: n = 791 female, n = 928 male)^2^ Source: PriCarenet network, Heidelberg University Hospital
^1^ For operationalisation, see Annex Table 1
^2^ Prevalences are colour-coded: blue stands for higher prevalences and light blue to white for lower prevalences (see colour scale). In principle, it is also possible to create a morbidity profile that includes multiple institutions.

The morbidity profile of this facility shows a predominantly primary care typical spectrum (Figure 4). Among adult patients, the morbidities with the highest prevalence are respiratory diseases (ICD-10: J00–J99) (female: 19.8%, male: 28.4%), musculoskeletal disorders (ICD-io: M00–M99) such as back pain (female: 14.3%, male: 19.8%), infectious diseases (ICD-10: A00–B99) (female: 14.0%, male: 21.1%), and diseases of the digestive system (ICD-10: K00–K99) (female: 14.3%, male: 16.2%). With a prevalence of 21.7%, conditions concerning pregnancy, childbirth and the postnatal period (ICD-10: O00–O99) are of particular relevance for female adult patients. Among children, diseases of the respiratory tract (female: 36.3%, male: 41.5%) are most prominent, with infectious diseases also a common condition among this group (female: 17.4%, male: 17.1%). While infectious diseases overall occur frequently, notifiable infectious diseases are comparatively rare among adults (female: 2.4%, male: 5.0%) and children (female: 0.5%, male: 1.3%).

Beyond the primary care spectrum, the data demonstrate that healthcare needs also extend to mental disorders (ICD-10: F00–F99) as well as consequences of external causes (ICD-10: S00–T98). Mental and behavioural disorders were diagnosed in 15.1% of men and 8.8% of women over the entire period. However, mental illnesses were also identified among girls (5.6%) and boys (6.5%). The morbidity indicator’ consequences of external causes’ includes injuries, burns and other conditions resulting from external causes such as accidents, assaults or operations of war. Men are particularly frequently affected, with a prevalence of 12.7%.

**Figure 5:**
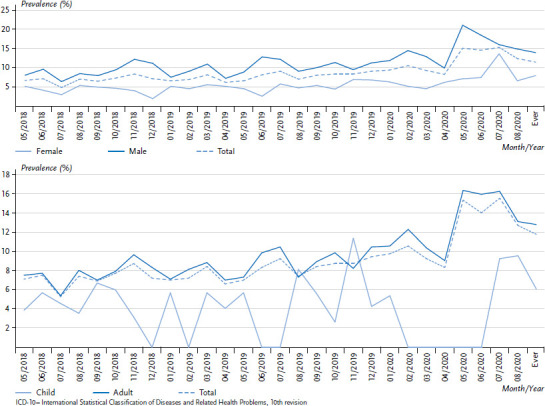
Prevalence of the indicator ‘mental and behavioural disorders’ (ICD-10: F00–F99), by sex (above) and age (below) for a sample facility (as a percentage of patients, n = 4,771 female, n = 6,798 male, n = 9,853 adults, n = 1,726 children) Source: PriCarenet network, Heidelberg University Hospital

**Annex Figure 1:**
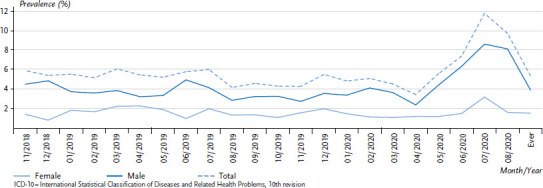
Prevalence of the indicator ‘mental and behavioural disorders’ (ICD-10: F00–F99, as a percentage of residents) for a sample facility Source: PriCarenet network, Heidelberg University Hospital

**Annex Figure 2:**
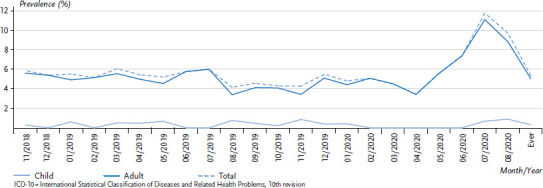
Prevalence of the indicator ‘mental and behavioural disorders’ (ICD-10: F00–F99, as a percentage of residents) for a sample facility Source: PriCarenet network, Heidelberg University Hospital

In principle, monthly analyses of absolute case numbers and prevalences for all morbidity indicators are available locally at the facility level. As an example, Figure 5 shows the monthly prevalence (based on the total number of people who received treatment) for ‘mental and behavioural disorders’ by sex and age over time. It demonstrates a particularly notable increase in prevalence from 8.2% in April 2020 to 15.1% in May 2020, which is mainly due to a doubling of the prevalence from 10.1% to 20.9% among male patients (Figure 5). In order to determine whether this increase can be explained by a fluctuation in the number of people living in the facility, a further analysis was conducted using this figure as the denominator. This analysis also identified the same pattern over time (Annex Figure 1 and Annex Figure 2).

### 3.2 Examples of results from a cross-facility analysis

**Figure 6:**
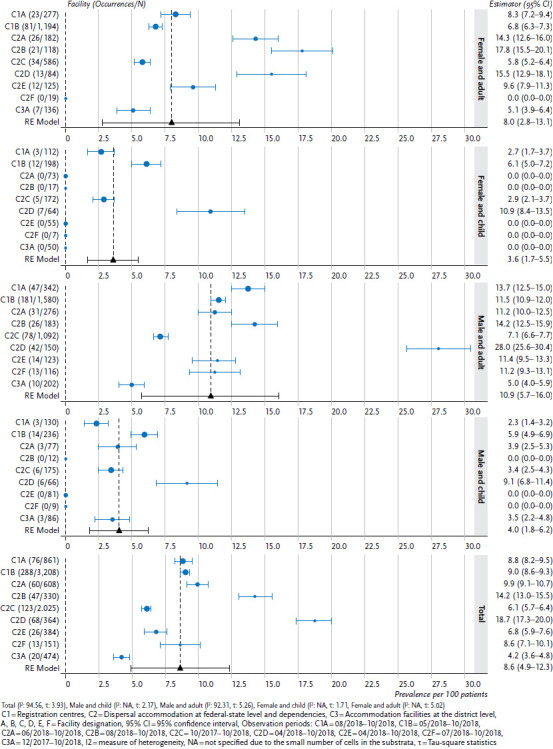
Mental and behavioural disorders (ICD-10: F00–F99) (as a percentage of the individuals who received treatment, adults: n = 2,721 female, n = 4,064 male, children: n = 748 female, n = 872 male) Source: PriCarenet network, Heidelberg University Hospital

Cross-facility analyses, particularly of morbidity indicators, can be conducted at regular intervals and the results are made available to the participating facilities for benchmarking purposes via the reporting platform. As an example, figure 6 shows the pooled prevalence of the morbidity indicator ‘mental and behavioural disorders’ for the period ranging from the introduction of RefCare© until November 2018 for eleven facilities (Figure 6). The meta-analysis found an 8.6% pooled prevalence of mental illnesses. Mental illnesses are mainly diagnosed among adults (women: 8.0%, men: 10.9%); the prevalence is 3.6% among girls and 4.0% among boys. Overall, the analysis reveals vast differences in the prevalence of mental illnesses between facilities.

## 4. Discussion

The PriCare project demonstrates that automated and timely health monitoring in reception centres for asylum seekers that is feasible and respects data protection regulations can be achieved through the innovative approach of networked distributed computing (the ‘hive approach’). For the first time, healthcare providers in reception centres for asylum seekers can work with an electronic medical records system that is tailored to their specific setting and provides for structured recording and analysis of morbidity, healthcare utilisation and other healthcare indicators. The facilities can now regularly analyse their routine medical data on-site without the need for specialist knowledge about statistical methods, compare their results with other facilities, and thus base healthcare planning on a solid data foundation. This approach also generates a body of data that can be used to aid political decision-makers and to support needs-based healthcare provision. However, if this is to be successful, the results not only need to be communicated in an appropriate manner, there also needs to be a willingness among politicians to consider data on healthcare needs in their decisions about healthcare provision in reception centres.

The results presented here demonstrate that the patients receiving care in the sample facilities exhibit a largely primary care-typical morbidity profile. At the same time, the results also highlight particular needs in terms of mental illness and consequences of external causes. Regarding mental health needs, the results underscore a high degree of heterogeneity between facilities, which may be due to the type of facility, the range of care available, and a facility’s particular demographic. The results also suggest that demographic aspects such as country of origin and the prevalence of individual diseases change dynamically, which underscores the need for continuous health monitoring.

This need is also clear from the noticeable decline over time in the indicator ‘mental and behavioural disorders’. Between April and May 2020, the respective facility was placed in quarantine for 16 days due to confirmed SARS-CoV-2 infections and all leisure and social support programmes were consequently cancelled. The decline remains stable even after fluctuations in the total number of occupants are taken into account, which means that changes in the total number of people living in the facility do not suffice as an explanation. However, nor does this descriptive analysis of the data demonstrate any clear association between what was happening in the facility during this period and an increased prevalence of mental illness. Instead, this increase could be due to random fluctuations over time or differences in coding practice resulting from personnel changes. Still, the results clearly illustrate the usefulness of health monitoring in this context: the descriptive time trend reveals a need for specific in-depth analyses that apply more complex procedures. This would involve examining possible associations in individual facilities and across facilities between, for example, measures put in place to contain the pandemic and the prevalence of psychological stress. Similar studies could be undertaken on the other morbidity and healthcare indicators, such as for vaccinations against influenza viruses and seasonal changes in the prevalence of respiratory infections.

In principle, this approach faces the typical limitations associated with the use of routine medical data [14]. These include issues of completeness, missing data, objectivity, reliability and, consequently, the validity of the content of the coded and documented data [14]. Since the monthly query of numbers of inhabitants has not proven practicable everywhere, the total number of patients (the outpatient population) has typically been used as the denominator. In periods with lower population flows, the figures for the total number of patients and inhabitants are closer to each other than during periods with greater levels of fluctuation. In the future, the higher discrepancy in the denominators during periods with a higher level of fluctuation could be accounted for with adjustment factors; however, these have yet to be developed empirically. In addition, heterogeneous coding behaviour leads to variance between and within facilities. Although this can be accounted for partially by using statistical methods, such as random effects models for the meta-analyses, the results are still not comparable to those gained from standardised primary studies such as health monitoring surveys.

Other challenges arise with the internal validation of diagnoses, especially when it comes to differentiating between suspected and confirmed diagnoses, as these differences are not always recorded. In addition, the spectrum of medical services provided and the function of the respective facilities have an impact on the range of diagnoses that will be recorded. It is fair to assume that illnesses that require specialised diagnostics will go underreported, not least because of the restricted entitlement to treatment set out in the Asylum Seekers Benefits Act. This becomes especially clear through comparisons between the prevalence of mental illnesses reported here and the prevalence identified using survey-based approaches (see also the article Monitoring the health and healthcare provision for refugees in collective accommodation centres in this issue of the Journal of Health Monitoring). Moreover, the morbidity profile in protective facilities housing asylum seekers with special needs can be expected to differ from that found in registration centres, where people remain for a very short period of time. Therefore, additional information about the context and the facility itself are important in order to adequately conduct and interpret cross-facility meta-analyses. In the PriCare project, the research network fulfils this purpose by providing a forum for the context-specific interpretation of the results.

However, the hive approach offers a resource-saving approach to ongoing, timely and comprehensive health monitoring without the added burden of data collection. To compensate for the limitations associated with the secondary use of routine medical data, the routine health monitoring could be supplemented with survey-based approaches undertaken at longer intervals (e.g. every three to five years) (see also the article Monitoring the health and healthcare provision for refugees in collective accommodation centres in this issue of the Journal of Health Monitoring).

In comparison to other efforts to utilise routine medical data from refugee camps in Europe to analyse health and healthcare parameters, too, the hive approach has significant advantages. For example, routine medical data from reception centres in Denmark and the Netherlands have been used in research. However, these analyses are based on centrally-stored routine medical data, and do not provide for regular analyses; as such, they are selective and guided by the interests of individual researchers [15, 16]. Therefore, these approaches are only partly suitable for timely, data protection-compliant health monitoring. Other approaches are based on the introduction of e-health systems, such as the system operated by the UN Relief and Works Agency (UNRWA), which is responsible for refugees from Syria, Lebanon, the West Bank and the Gaza Strip [17]. Research has also been undertaken using electronic patient files, such as those from the International Organization for Migration as part of the e-PHR project [18] and the electronic files held in Germany on asylum applicants [9]. However, these web-based applications for recording routine medical data use central (‘cloud-based’) data storage and are therefore associated with the risks and challenges of maintaining and protecting a database with sensitive personal data from a vulnerable population group (see also [9]). Distributed computing avoids these problems, while still enabling health monitoring to be conducted using individual-level data.

As technology development, methods, processes and the structures required for the PriCare project were funded by the Federal Ministry of Health, the next challenge is consolidating these structures and ensuring that they remain in place in the long term. Bilateral, non-commercial utilisation and licensing agreements have been concluded with most facilities within the network, and this should enable the project to continue for the time being. However, optimal long-term use of the infrastructure and procedures established by the PriCare project would require their expansion to all accommodation facilities in all federal states and, above all, the development of sustainable health reporting. This would enable the health of and the healthcare provided to refugees to be monitored in all accommodation facilities throughout the country and would, therefore, permanently close existing data gaps.

The hive approach can also be used in areas of health services research beyond the healthcare provision in reception centres for asylum seekers. The federal health system, with its fragmented healthcare provision and data landscape, poses similar challenges to those described above when using routine medical data for health monitoring and research. The application of distributed computing, therefore, could bean important tool for the Medical Informatics Initiative [17] as well as for prompt analysis and reporting of notifiable diseases. The limitations faced by conventional approaches such as the use of centralised databases or federal reporting systems could thus be avoided. The hive approach also avoids time lags of reports of notifiable diseases filed with district-level public health services reaching the federal level. The COVID-19 pandemic has shown that reporting lags of just a few days can be of great importance for supra-regional health monitoring, evidence-based political decision-making and the broader public. However, distributed computing requires a standardised or an at least interoperable database structure across participating institutions that enables standardised scripts to be run on-site. This produces comparable, anonymised results that can then be made available for meta-analysis.

In cases like the implementation of the hive approach in reception centres for asylum seekers, where routine medical data are analysed for all patients without obtaining written consent, additional issues need to be addressed in order to guarantee data-protection compliance. In particular, a decision-making body is required to ensure that medical service providers have a say regarding the indicators used in the analyses of the local routine data. Distributed computing could be implemented in other areas of health with great potential once these foundations have been put in place.

In addition to the results from the statistical analysis, developing the infrastructure needed to implement the ‘hive approach’ in the heterogeneous settings of reception centres has led to positive side-effects. The implementation of standardised medical records that comply with data-protection regulations when transferring patient records between facilities, and provides customised outpatient administrative functions, contributes towards reducing the workload for its users and improving healthcare provision. Furthermore, the project encourages facilities to consider legal, administrative and organisational aspects relating to the protection of medical records in the often precarious and fragmented settings of care provision, and to clarify where data protection responsibilities lie within their facilities.

Finally, in addition to developing the indicators used for health monitoring, the PriCarenet research network has also proven an important forum for sharing experiences between the participating institutions. This encourages members to discuss experiences, challenges and best practices. The interpretation of the results from health monitoring as well as discussion within the research network about possible implications for healthcare provision and planning also contribute to the further improvement of healthcare provision in reception centres for asylum seekers.

## Key statements

- Data on healthcare provision to refugees and asylum seekers in reception centres is essential for individual healthcare and needs-based care planning.
- Data are not yet systematically available for healthcare planning, research and reporting.
- A lack of routine medical data and differences between medical records currently preclude health monitoring across multiple regions.
- Digitisation of routine medical data is essential for the systematic health monitoring of refugees.
- Networks and distributed computing enable timely and data-protection compliant health monitoring to be undertaken in reception centres.
- In addition to primary medical conditions, asylum seekers in reception centres often require treatment due to mental illnesses and consequences of external causes, such as accidents, assault, or operations of war.
- Consolidating and integrating decentralised analyses into data collection and evaluation structures would close existing data gaps.

## Figures and Tables

**Table 1:** The current health and care indicator set (n = 64) Source: PriCarenet network, Heidelberg University Hospital

Indicator
**Population**
Total population
Patients
**Morbidity**
Multimorbidity
Disabilities, by diagnosis
Diseases of the skin and subcutaneous tissue, by diagnosis
External causes of morbidity and mortality, by diagnosis
Consequences of external causes
Frequent outpatient diagnoses in accordance with ICD-10-GM
Diseases of the digestive system, by diagnosis
Diseases of the blood-forming organs, by diagnosis
Certain infectious and parasitic diseases
Notifiable infectious diseases
Infectious agents that are resistant to certain antibiotics or chemotherapy
Diseases of the circulatory system, by diagnosis
Hypertension
Body Mass Index
Hypercholesterolemia
Endocrine, nutritional and metabolic diseases, by diagnosis
Diabetes mellitus
Diseases of the musculoskeletal system and connective tissue, by diagnosis
Neoplasms, by diagnosis
Diseases of the nervous system, by diagnosis
Diseases of the ears and mastoid process, by diagnosis
Diseases of the eyes and adnexa, by diagnosis
Certain conditions originating in the perinatal period by diagnosis
Events related to pregnancy, childbirth and the puerperium
Frequency of pregnancies
Mental disorders and behavioural problems, by diagnosis
Therapy with psychotherapeutic medication
Prescription benzodiazepines
Diseases of the respiratory system, by diagnosis
Diseases of the genitourinary tract, by diagnosis
**Quality of care**
Prescriptions of antibiotics among adults
Ambulatory care sensitive hospitalisations among adults
Ambulatory care sensitive hospitalisations among children
DPT vaccination in children <1 year
DPT vaccination in children 1–5 years
Documentation of vaccination history
Primary immunisation against diphtheria, tetanus, polio started
Basic immunisation against diphtheria, tetanus, polio completed
Frequency of internally performed STIKO vaccinations
Frequency of externally performed STIKO vaccinations
Patients diagnosed as HIV positive undergoing therapy
Consultations where there was a language barrier
Approved reimbursement requests
Diabetes mellitus treatment
Metabolic complications in diabetes mellitus
**Healthcare processes**
Total number of patient visits
Average number of visits per patient
Healthcare services utilisation per inhabitant
Ten most common reasons for seeking medical advice
Referrals to outpatient, specialist medical facilities
Referral to in-patient facilities
Factors that affect health and lead to healthcare utilisation
Potentially health-endangering incidents (critical incidents)
**Syndromic surveillance**
Acute respiratory infection
Chronic cough
Fever and rash
Meningitis/encephalitis
Gastroenteritis
Bloody diarrhoea
Skin parasitosis
Fever and bleeding
Acute jaundice

ICD-10-GM = International Statistical Classification of Diseases and Related Health Problems, 10th revision, German Modification,

DPT = combination vaccine against diphtheria, pertussis and tetanus, STIKO = Standing Committee on Vaccination

## Appendix

**Annex Table 1:** Overview of the operationalisation of the indicators Source: Own table

Indicator	Operationalisation (ICD-10 codes)
Disabilities	H54, R47, H90–H91, H80–H82, Q71–Q73, M20–M21, Z89, G82, F06–F07, 168, P91, F7, F1
Diseases of the skin and subcutaneous tissue	L00–L99
External causes of morbidity and mortality by diagnosis	V01–Y84
Consequences of external causes of morbidity and mortality	S00–T98
Digestive system diseases	K00–K99
Diseases of the blood and the blood-forming organs	D50–D90
Infectious and parasitic diseases	A00–B99
Notifiable infectious diseases	B30.0, B30.1, A05.1, A23.0, A23.1, A23.3, A23.8, A23.9, A04.5, A92.0, A00, A81.0, A97, A36, A98.4, A04.4, B67, A04.3, A75.0, A84.1, A95, A07.1, A41.3, A49.2, G00.0, J09, J14, J20.1, P23.6, A98.5, B15, B16, B17.1, B18.2, B19, B16.0, B16.1, B17.0, B17.2, B17.8, B20-B24, D59.3, M31.1, J09, J10, J11, A37, A07.2, A96.2, A68.0, A48.1, A48.2, A30, A27, A32, P37.2, B50-B54, A98.3, B05, A39, A41.0, A49.0, G00.3, P36.2, A22, B26.8, B26.9, A08.1, A70, A01.1, A01.2, A01.3, A01.4, A20, A80, A78, A08.0, P35.0, B06.8, B06.9, A0, A03, A50, A53, A82, Z20.3, P37.1, B75, A15–A19, P37.0, O98.0, A21, A01.0, A92.0, A92.4, A96, A98.0, A98.1, A99, B02, P35.8, A04.6
Infectious agents that are resistant to certain antibiotics or chemotherapy drugs	U80–U85
Circulatory system diseases	100–199
Hypertension	110–115 (or vital parameters: blood pressure > 140/90 mmHg)
Body Mass Index (BMI)	E65–E68
Hypercholesterolemia	E78
Endocrine and Metabolic Diseases	E00–E9
Diabetes mellitus	E10–E14
Diseases of the musculoskeletal system and connective tissue	M00–M99
Neoplasms by diagnosis	C00–D48
Malignant neoplasms	C00–C97
Nervous system diseases	G00–G99
Diseases of the ear and mastoid process	H60–H99
Diseases of the eyes and appendages	H00–H59
Disorders originating in the perinatal period	P00–P96
Events related to pregnancy, childbirth and the puerperium	O00–O99
Abortion	O00–O08
Mental and behavioural disorders	F00–F99
Therapy with psychotherapeutic medication	ATC codes: N05 (antipsychotics, anxiolytics), N06A, N06B, N06C (antidepressants, psychostimulants, herbal psychotropic drugs), N07BB (drug for alcohol addiction treatment)
Prescription benzodiazepines	ATC codes: N05BA (anxiolytics) or N05CD (hypnotics)
Respiratory system diseases	J00–J99
Diseases of the genitourinary system	N00–N99

ATC = Anatomical-Therapeutic-Chemical Classification System for Medicinal Products,

ICD-10 = International Statistical Classification of Diseases and Related Health Problems, 10th revision
